# NREM Sleep Oscillations and Brain Plasticity in Aging

**DOI:** 10.3389/fneur.2012.00176

**Published:** 2012-12-14

**Authors:** Stuart Fogel, Nicolas Martin, Marjolaine Lafortune, Marc Barakat, Karen Debas, Samuel Laventure, Véronique Latreille, Jean-François Gagnon, Julien Doyon, Julie Carrier

**Affiliations:** ^1^Department of Psychology, Université de MontréalMontréal, QC, Canada; ^2^Functional Neuroimaging Unit, Centre de Recherche de l’Institut Universitaire de Gériatrie de MontréalMontréal, QC, Canada; ^3^Center for Advanced Research in Sleep Medicine, Hôpital du Sacré-Cœur de MontréalMontréal, QC, Canada

**Keywords:** aging, sleep, NREM, declarative memory, procedural memory, cognition, slow waves, sleep spindles

## Abstract

The human electroencephalogram (EEG) during non-rapid eye movement sleep (NREM) is characterized mainly by high-amplitude (>75 μV), slow-frequency (<4 Hz) waves (slow waves), and sleep spindles (∼11–15 Hz; >0.25 s). These NREM oscillations play a crucial role in brain plasticity, and importantly, NREM sleep oscillations change considerably with aging. This review discusses the association between NREM sleep oscillations and cerebral plasticity as well as the functional impact of age-related changes on NREM sleep oscillations. We propose that age-related reduction in sleep-dependent memory consolidation may be due in part to changes in NREM sleep oscillations.

## Slow-Wave NREM Sleep Oscillations

Non-rapid eye movement (NREM) sleep is characterized by different degrees of cortical synchronization, from lower in lighter sleep stages (1 and 2) to higher in deeper slow-wave sleep (SWS) stages. In mammals, more time spent awake results in higher cortical synchronization in NREM sleep, whereas synchronization dissipates as sleep progresses (Achermann et al., 1993). Cortical synchronization during NREM sleep is characterized by slow waves (SW). At the cellular level, SW have two phases: hyperpolarization [surface electroencephalogram (EEG) negative], during which cortical neurons are mostly silent (OFF period); and depolarization (surface EEG positive), during which most cortical neurons fire intensively (ON period; Steriade, 2006; Csercsa et al., 2010). Animal studies have demonstrated that, under high homeostatic sleep pressure, short periods of intense cortical activity (ON periods) alternate frequently with relatively long periods of neuronal silence (OFF periods). Conversely, under low homeostatic sleep pressure, long ON periods are interrupted by sporadic, short OFF periods (Vyazovskiy et al., 2009). Greater homeostatic sleep pressure is associated with higher SW density and amplitude, faster SW frequency (Hz), and steeper slope between negative and positive phases (Esser et al., 2007; Riedner et al., 2007; Bersagliere and Achermann, 2010; Carrier et al., 2011; Mongrain et al., 2011).

Electroencephalogram SW originate more frequently in prefrontal–orbitofrontal regions and travel in an anteroposterior direction (Massimini et al., 2004). Source modeling has shown that SW are more likely to originate in the insula and the cingulate gyrus, but often involve frontal areas and the precuneus (Murphy et al., 2009). Recent fMRI studies have confirmed that SW are associated with activation of the brainstem, inferior frontal gyrus, posterior cingulate cortex, and precuneus, which are important for sleep maintenance and cerebral plasticity (Dang-Vu et al., 2008).

## Slow Waves and Cerebral Plasticity

Increasing evidence suggests that specific brain areas that are recruited for a particular task may require more slow-wave activity (SWA) than other brain areas (Kattler et al., 1994; Huber et al., 2004). According to the synaptic homeostasis hypothesis, cerebral plastic processes during wakefulness produce a net increase in synaptic strength, reducing the capacity for further potentiation. SWA during NREM sleep is associated with the downscaling of synaptic strength, which then allows for further synaptic potentiation and learning to occur following sleep (Tononi and Cirelli, 2006). Accordingly, waking behaviors associated with synaptic potentiation would be expected to increase SW, whereas waking behaviors associated with synaptic depression would be expected to reduce SW (Hanlon et al., 2009). In support of this hypothesis, somatosensory stimulation (Kattler et al., 1994) and training on a motor adaptation task (Huber et al., 2004) increased SWA the following night. Additional support for the synaptic homeostasis hypothesis in humans is supported by studies that applied TMS to manipulate neural excitability immediately prior to sleep (Huber et al., 2007, 2008; Massimini et al., 2009). Results showed that TMS-evoked increases in cortical responsiveness (potentiation) prior to sleep enhanced local SWA at central derivations, whereas TMS-evoked decreases in cortical responsiveness (depression) reduced SWA locally during subsequent sleep. These findings provide compelling evidence in support of the synaptic homeostasis hypothesis (Huber et al., 2007, 2008) and the potential role of SW in brain plasticity (Tononi and Cirelli, 2006).

## Characteristics of Sleep Spindle Oscillations

Sleep spindles appear on the EEG as transient waxing and waning oscillations recurring approximately once every 3–10 s (Steriade, 2005). Two types of spindles have been described, distinguished by their frequency, scalp topography, and associated neural correlates identified using fMRI (Schabus et al., 2007). Slow spindles (∼11–13 Hz) predominate frontal derivations, whereas fast spindles (∼13–15 Hz) are more prominent at centro-parietal derivations (De Gennaro and Ferrara, 2003). Spindles reflect oscillatory activity in thalamocortical (TC) networks during NREM sleep, and may be associated with loss of perceptual awareness and consolidation of memory traces (Steriade, 2006). On the one hand, spindle oscillations have the intrinsic property to hyperpolarize TC cells through recurrent inhibition of reticular thalamic activity. This inhibitory spindle activity prevents afferent signals from being transmitted to the cortex, thus reducing cortical responsiveness to external stimulation during sleep (Steriade, 1994; Dang-Vu et al., 2011). On the other hand, post-inhibitory rebound firing of TC cells causes the depolarization of cortical pyramidal neurons through rhythmic excitatory spike bursts. Cortical involvement is crucial in mediating spindle duration, as recently observed by intracellular recordings and computational models (Bonjean et al., 2011). Corticothalamic (CT) feedback can not only trigger spike bursts in reticular thalamic neurons to initiate a spindle oscillation, but it also can terminate the spindle by desynchronizing the thalamic network through depolarization of TC cells. Thus, spindle duration can be maintained by a synchronized spiking activity of the TC system, but is interrupted when thalamic and cortical firing fall out of phase.

## Sleep Spindles and Synaptic Plasticity

The highly recurrent and coherent firing pattern associated with sleep spindles may promote plastic changes underlying learning and memory consolidation (Steriade and Timofeev, 2003). Indeed, spindle activity creates the ideal conditions for massive Ca^2+^ entry into depolarized dendrites of cortical neurons, a molecular event involved in synaptic plasticity and long-term potentiation (LTP; Sejnowski and Destexhe, 2000). In fact, LTP was successfully induced by trains of spindle activity in the rat somatosensory cortex (Rosanova and Ulrich, 2005), and the induction of LTP by tetanization of the rat corpus callosum increased the reliability of evoked spindle oscillations (Werk et al., 2005). Human neuroimaging studies have revealed that both fast and slow spindles are associated with increased hemodynamic responses in the thalamic nuclei and paralimbic areas (Schabus et al., 2007). Slow spindles are associated with greater cerebral activation in the superior frontal gyrus, whereas fast spindles recruit several cortical regions usually involved in sensorimotor processing as well as the hippocampus and mediofrontal areas. In addition, evidence from rodents has shown that prefrontal cortical spindles are temporally coordinated with high-frequency ripples in the hippocampus (Sirota et al., 2003). Since sleep spindles have been implicated in memory consolidation for hippocampal-dependent memory (see Fogel and Smith, 2011, for review), it is tempting to speculate that the temporal relationship between spindles and ripples may be a reflection the hippocampal-neocortical dialog thought to be required for memory consolidation (Siapas and Wilson, 1998). Taken together, these findings suggest that sleep spindles are associated with neurophysiological processes underlying brain plasticity.

## Age-Related Changes in NREM Sleep Oscillations

Non-rapid eye movement sleep synchronization changes considerably with age, with a substantial reduction in SWS, an increase in lighter NREM sleep stages, and significant decreases in SWA (spectral power between 0.5 and 4 Hz) and sigma (13–14 Hz) activity during NREM sleep, beginning as early as middle age (Carrier et al., 2001; Landolt and Borbély, 2001; Darchia et al., 2007). Previous studies have shown the greatest age-related decreases in the spectral power of delta frequencies in frontal derivations (Munch et al., 2004; Robillard et al., 2010). Recently, we evaluated cortical mechanisms of NREM synchronization by characterizing changes in automatically detected SW in a large sample of young and middle-aged subjects (Carrier et al., 2011). Compared to young adults, older individuals had both lower SW amplitude and lower SW density, especially in prefrontal/frontal brain areas, where they most frequently originate (Massimini et al., 2004). In addition, older subjects had lower SW slope and longer SW positive and negative phases compared to young subjects. These age-related differences suggest that cortical neurons take more time to synchronously enter SW hyperpolarization and depolarization phases. This age-related reduction in slow activity during NREM sleep may have a negative impact on brain plasticity and sleep-dependent memory consolidation.

In addition to reduced SW during NREM sleep, spindle density, amplitude, and duration are reduced in older compared to young subjects (Guazzelli et al., 1986; Wei et al., 1999; Crowley et al., 2002), and this decline has been shown to be progressive with age (Principe and Smith, 1982; Nicolas et al., 2001). Yet spindle frequency shows a slight increase with age (Principe and Smith, 1982; Wei et al., 1999; Nicolas et al., 2001; Crowley et al., 2002). Spindle dynamics over the course of the night also change with age. While young subjects typically show increased sigma activity and spindle density from the beginning to the end of the night (Jankel and Niedermeyer, 1985; Aeschbach and Borbély, 1993), older subjects do not show this trend, or show it to a lesser extent (Guazzelli et al., 1986; Landolt et al., 1996; Wei et al., 1999).

Most studies exploring the effects of age on sleep spindles have restricted their analysis to a single EEG scalp derivation. Yet spindles are known to be topographically heterogeneous (Zeitlhofer et al., 1997). In fact, Nir et al. (2011) recently demonstrated that the majority of spindles occur independently across brain regions: most spindles appeared simultaneously in only a minority of brain areas, suggesting local rather than global modulation. Consistent with Nir et al. (2011), we reported that age effects differed across scalp location (Martin et al., 2013). Age-related decreases in spindle density and amplitude were most prominent at anterior derivations, whereas shorter duration was maximal at posterior derivations. Surprisingly, there were no age-related differences in spindle frequency at any derivations. It remains to be tested whether an age-related reduction in sleep spindle activity may have a negative impact on sleep stability (Dang-Vu et al., 2010), and whether this may underlie reduced age-related sleep-dependent memory consolidation.

## Role of NREM Sleep Oscillations in Declarative Learning

The contributions of sleep to learning, memory, and brain plasticity have been studied for over 30 years (Smith et al., 1974; for a review, see Smith, 1985, 1996; Maquet, 2001; Walker, 2005; Stickgold and Walker, 2007; Diekelmann and Born, 2010). However, it remains a contentious topic (Stickgold and Walker, 2005). Indeed, it is unclear which sleep stages support the enhancement and/or consolidation of different memory systems.

Numerous recent studies propose the role of NREM sleep oscillations in declarative learning. It has been hypothesized that SW-dependent downscaling benefits memory by eliminating weak synapses (i.e., noise), thereby improving the signal-to-noise ratio in the synapses that were strongly potentiated during prior wakefulness (Tononi and Cirelli, 2006). According to another dominant hypothesis, declarative memory consolidation during sleep would occur during SWS via repeated reactivations (replay; see Bergmann et al., 2012; Diekelmann and Born, 2010, for recent reviews). This replay would benefit memory by transferring new memories from the hippocampus to the neocortex via hippocampal–neocortical dialog. In addition, Buzsáki (1989, 1998) has provided an alternative, but complementary viewpoint largely based on work done in rats. He suggests that during SWS, potentiated neurons of the hippocampus communicate with the neocortex via sharp-wave bursts. This communication is thought to underlie the hippocampal-neocortical transfer of information during sleep, leading to memory consolidation.

Studies suggest the key role of hippocampal/neocortical reactivation during early NREM in the consolidation of hippocampal-dependent memories, such as declarative memory (Peigneux et al., 2004; Rasch et al., 2007). Compared to an equivalent period of wake, post-learning sleep has been shown to enhance declarative memory task retention (e.g., word pair retention; Tucker et al., 2006; Gais et al., 2007) and increase hippocampal activation during recall (Gais et al., 2007). In rats, the pattern of hippocampal place cell firing observed during spatial learning is reproduced during subsequent periods of SWS (Wilson and McNaughton, 1994), suggesting that memory reactivation may occur during SWS. In humans, Peigneux et al. (2004) observed that brain regions (including the hippocampus) that are active during virtual maze navigation are also active during SWS, suggesting that SWS may play a similar role for spatial memory reactivation in humans. Furthermore, declarative memory benefits mainly from sleep periods rich in SWS rather than rapid eye movement (REM) sleep (Plihal and Born, 1999; Gais and Born, 2004; Drosopoulos et al., 2005). A recent study (Rasch et al., 2007) has shown that spatial declarative memory can be enhanced when a contextual odor cue during learning is presented again during SWS. No enhancement was observed in the vehicle-only control condition or when the odor was re-presented during REM sleep. They concluded that the cue served to reactivate the memory trace during SWS, inducing enhanced SWS-dependent memory consolidation. Moreover, several studies have reported positive associations between the characteristics of NREM sleep including SWA, SW, and spindle oscillations and greater declarative memory retention after sleep (Clemens et al., 2005; Tucker and Fishbein, 2008; Tamminen et al., 2010; Van Der Werf et al., 2011; Wilhelm et al., 2011). However, several of these studies lacked a control night to distinguish state-like associations (spindle enhancement after learning and memory performance) from trait-like associations (inter-individual differences in cognitive abilities related to individual differences in spindles). It is necessary to consider inter-individual differences in spindle activity when investigating their night-to-night changes in response to learning, since spindles are associated with rather stable cognitive traits such as intellectual ability (Schabus et al., 2006; Fogel et al., 2007a). Nonetheless, several studies have clearly demonstrated that declarative learning prior to sleep increases SW and spindle oscillations, which are associated with overnight memory retention (Gais et al., 2002; Schabus et al., 2004, 2008; Schmidt et al., 2006; Molle et al., 2009). Moreover, boosting SW with transcranial direct current stimulation (TDCS) during sleep enhances verbal declarative memory retention (e.g., learning word pair associations; Marshall et al., 2006), and reducing SWA and slow sigma power during NREM sleep with TDCS reduces verbal declarative memory retention (Marshall et al., 2011), suggesting a causal relationship between SW and declarative memory. Thus, taken together, these results suggest that NREM sleep plays an important, if not a crucial role for declarative memory consolidation. Moreover, specific aspects of NREM sleep such as SW and spindles are correlated with memory consolidation and increase in a learning-dependent manner following declarative learning. Thus, suggesting that these events may play an active role in memory consolidation processes.

## The Impact of Age-Related Changes in Sleep on Declarative Memory

Currently, there is no clear consensus on the impact of aging on sleep-dependent declarative memory consolidation. One study identified reduced sleep-related consolidation of verbal memory in older subjects, associated with an age-related decrease in early-night SWS (Backhaus et al., 2007). Other studies found no age-related differences in sleep-dependent consolidation of verbal declarative memory (Aly and Moscovitch, 2010; Wilson et al., 2012). Discrepancies between these results may be attributable to statistical power issues and confounding factors, such as circadian effects on initial encoding and retrieval, and importantly, their interaction with age. Future studies should also use objective sleep measurements.

To our knowledge, only three studies have assessed whether NREM sleep oscillations are associated with declarative overnight memory retention in older participants. Higher SWA was linked with better overnight declarative memory retention in both healthy elderly adults and patients with mild cognitive impairment (Westerberg et al., 2012), whereas higher sleep spindle density was associated with better overnight visuospatial memory retention in older women (Seeck-Hirschner et al., 2012). A study in Alzheimer patients found no association between spindles and overnight memory retention, but instead found a positive relationship between memory performance prior to sleep and spindle intensity in subsequent sleep (Rauchs et al., 2008). However, these studies did not control for baseline sleep to distinguish between state-like associations versus trait-like associations. Nevertheless, another study has assessed a clear state-like association between SWA and memory performance in healthy elderly patients, by experimentally reducing SWA using acoustic neurofeedback. This procedure was found to decrease the encoding of novel declarative information in older subjects (Van Der Werf et al., 2011). Taken together, these results suggest that NREM sleep spindles and SWA are related to declarative memory encoding and retention in older adults. The impact of age-related changes in sleep, and specifically the changes in NREM sleep oscillations on sleep-dependent declarative memory consolidation remains to be clarified.

## NREM Sleep Oscillations and Procedural Learning

Consolidation of various procedural skills (e.g., finger movements, gross movements involving target tracking, dexterity, tracing, and fine motor skills) has been shown to benefit from a period of sleep, whether a daytime nap (Fischer et al., 2002; Korman et al., 2003, 2007; Backhaus and Junghanns, 2006; Milner et al., 2006; Nishida and Walker, 2007; Doyon et al., 2009) or overnight sleep (Fischer et al., 2002; Walker et al., 2002; Korman et al., 2003; Peters et al., 2007). Among the most consistent findings in support of the role of sleep in memory consolidation is a series of studies demonstrating a link between procedural memory and stage 2 (Smith and MacNeill, 1994; Fischer et al., 2002; Walker et al., 2003; Fogel and Smith, 2006; Fogel et al., 2007b) and SWS sleep (Huber et al., 2004; Fogel et al., 2007b). Even a short amount of stage 2 sleep from daytime nap was found to be correlated with the delayed gains in performance on a procedural task (Korman et al., 2007). Given that procedural learning is a broadly defined and heterogeneous category, it is important to first distinguish between the types of task demands, in order to understand the link between procedural learning and sleep states. On the one hand, there is evidence that task performance depends more on REM sleep when the task involves the acquisition of entirely new skills or rules, or when individuals are not very proficient during acquisition (Smith and Weeden, 1990; Buchegger et al., 1991; Smith, 1993; Plihal and Born, 1997; Smith and Smith, 2003). On the other hand, procedural learning appears to depend more on stage 2 sleep when the task is cognitively simple and involves the refinement of skills that individuals may have already acquired to some extent (Fischer et al., 2002; Walker et al., 2002; Fogel and Smith, 2006; Fogel et al., 2007b). A number of studies support this hypothesis, originally proposed by Smith et al. (2004). The first direct evidence for this dissociation (Peters et al., 2007) revealed that when subjects were trained on a rotary pursuit procedural task and then subdivided into “high-skill” and “low-skill” groups, post-training spindle density was correlated with performance in the high-skill but not the low-skill group. Conversely, post-training changes in REM density were correlated with performance in the low-skill but not the high-skill group. Although correlational, this dissociation suggests that the type of sleep involved in memory consolidation depends on not only task type or task demands, but perhaps the individual’s proficiency on the task.

Further support for this hypothesis comes from deprivation studies showing that stage 2 sleep disruption, but not REM deprivation in the last half of a night’s sleep, leads to impaired performance on the pursuit rotor, a simple procedural task (Smith and MacNeill, 1994). In addition, studies investigating the impact of procedural learning on subsequent sleep found increased stage 2 sleep duration following simple procedural learning (Fogel and Smith, 2006; Fogel et al., 2007b). Moreover, sleep spindles in stage 2 sleep increased following simple procedural learning involving gross motor skills (Fogel and Smith, 2006; Fogel et al., 2007b) and motor sequence learning (MSL; Walker et al., 2002). Not only does sleep spindle density increase after performing a simple procedural task, but the morphology of the spindle also changes: sleep spindle amplitude and duration were shown to increase, and the increase in spindle density persisted into SWS (Fogel et al., 2007b). Taken together, these results suggest that the brain responds to simple procedural learning by increasing sleep spindles in a variety of ways, collectively leading to massively increased spindle activity over the course of a night’s sleep. These changes are thought to reflect memory consolidation processes during sleep.

Initially, MSL relies on a variety of brain structures, including the cerebellar-cortical and striatal-cortical networks (for a review, see Doyon et al., 2003), and in some cases the hippocampus was found to be involved (Albouy et al., 2008). Once consolidated, procedural memory generally relies on a network of striatal-cortical structures (Doyon et al., 2003, 2009; Doyon and Benali, 2005). It was proposed that sleep spindles play a role in the transfer (or transformation) of information from brain structures involved in learning and in early consolidation of MSL (Walker et al., 2005). A recent study (Barakat et al., 2012) showed that spindle amplitude at frontal regions was correlated with offline gains in performance and changes in activity in the putamen over a sleep interval. These results provide some of the first direct evidence of the neural substrates involved in sleep-dependent MSL consolidation that is specifically related to spindle activity.

The role of sleep in motor adaptation tasks is less clear. While we showed that procedural consolidation was not sleep-dependent (Doyon et al., 2009), Huber et al. (2004) observed increased SWA during sleep after subjects were trained on a more complex motor adaptation task. The increased SWA was circumscribed to a region of the posterior parietal cortex known to be activated by this task (Ghilardi et al., 2000). Motor adaptation task complexity may also explain discrepancies between studies.

## The Impact of Age-Related Changes in Sleep on Procedural Memory

Age-related changes in sleep may have a negative impact on sleep-dependent procedural memory consolidation. In support of this hypothesis, Spencer et al. (2007) investigated the effect of a retention period filled with either sleep (or wake) on the performance of implicit and explicit versions of a MSL task in young and older subjects. They found that sleep-dependent gains in performance were observed in young but not in older subjects for both the implicit and explicit task versions of the task. In addition, Brown et al. (2009) has shown that sleep does not provide the same benefit to procedural memory consolidation in older adults as compared to young subjects. More specifically, they used an implicitly learned, cued version of the serial reaction time task to assess MSL for both sequence and random-order versions of the task. Surprisingly, increased learning was observed in older subjects during the training session as compared to young subjects. However, only young subjects showed gains in performance over an intervening retention sleep period. It is important to note that this result was not attributable to age-related differences in speed or baseline performance. While these two studies suggest that sleep provides a benefit to procedural memory consolidation in young but not in older individuals, the characteristics of sleep were not investigated. A related study by Peters et al. (2008) suggested that age-related changes in sleep spindles were correlated with sleep-dependent procedural memory consolidation in young but not older adults. They found that learning a pursuit rotor task increased sleep spindles in young subjects but not in older subjects. Furthermore, whereas both young and older subjects improved with practice on the pursuit rotor task, the magnitude of the improvement was greater for young subjects when tested 1 week after initial learning, suggesting that young subjects performance was consolidated to a greater extent than older individuals. Better performance in young subjects at acquisition was associated with larger increases in spindle density, but not in older individuals. These results suggest that the age-related reduction in sleep spindles may underlie age-related deficits in procedural memory consolidation, however this hypothesis remains to be directly tested.

## Conclusion

One of the most marked changes in sleep in elderly populations is a reduction of SW and sleep spindles. NREM sleep oscillations are related to the reactivation of recently learned material, cerebral plasticity, and synaptic homeostasis. We propose that an age-related reduction in sleep-dependent memory consolidation may be due in part to changes in NREM sleep oscillations. Further research is required where NREM sleep oscillations are causally manipulated, to better disentangle the role that sleep spindles and SW have for memory consolidation in older subjects. Spindles and SW are generated by the neural oscillation between hyperpolarized and depolarized phases, believed to play a crucial role in brain plasticity (Steriade, 2006). At present, there is ample evidence to suggest that spindles and SW are involved in the consolidation of both declarative and procedural learning. Thus, it is difficult to conclude whether they serve dissociable roles for different memory systems, or not. Future research should evaluate whether age-related changes in spindles and SW interfere differently with declarative and procedural learning.

## Conflict of Interest Statement

The authors declare that the research was conducted in the absence of any commercial or financial relationships that could be construed as a potential conflict of interest.
